# Enhancement of the Structure, Thermal, Linear/Nonlinear Optical Properties, and Antibacterial Activity of Poly (vinyl alcohol)/Chitosan/ZnO Nanocomposites for Eco-Friendly Applications

**DOI:** 10.3390/polym15214282

**Published:** 2023-10-31

**Authors:** E. M. Abdelfattah, H. Elzanaty, W. B. Elsharkawy, M. A. Azzam, Z. M. Elqahtani, S. Alotibi, M. Alyami, T. Fahmy

**Affiliations:** 1 Physics Department, College of Science and Humanities, Prince Sattam Bin Abdulaziz University, Alkharj 11942, Saudi Arabia; e.abdelfattah@psau.edu.sa (E.M.A.); w.elsharkawy@psau.edu.sa (W.B.E.); sf.alotibi@psau.edu.sa (S.A.); m.alyami@psau.edu.sa (M.A.); 2 Physics Department, Faculty of Science, Zagazig University, Zagazig 44519, Egypt; 3 Department of Basic Science, Faculty of Engineering, Delta University, Mansoura 11152, Egypt; dr.hesham_aly@yahoo.com; 4 Chemistry Department, College of Science and Humanities, Prince Sattam Bin Abdulaziz University, Alkharj 11942, Saudi Arabia; m.azzam@psau.edu.sa; 5 Department of Physics, College of Science, Princess Nourah Bint Abdulrahman University, P.O. Box 84428, Riyadh 11671, Saudi Arabia; 6 Polymer Research Group, Physics Department, Faculty of Science, Mansoura University, Mansoura 35516, Egypt; tfahmy5@mans.edu.eg

**Keywords:** PVA/Cs, ZnO NPs, thermal stability, bandgap, antibacterial activity

## Abstract

The preparation of poly (vinyl alcohol)/chitosan/ZnO (PVA/Cs/ZnO) nanocomposite films as bioactive nanocomposites was implemented through an environmentally friendly approach that included mixing, solution pouring, and solvent evaporation. The nanocomposite films were characterized using various techniques such as X-ray diffraction (XRD), Fourier-transform infrared (FT-IR) spectroscopy, differential scanning calorimetry (DSC), thermogravimetric analysis (TGA), and UV-Vis spectroscopy. The XRD study revealed the encapsulation of nanoparticles by the PVA/Cs blend matrix. The DSC results showed that the addition of ZnO NPs increased glass transition and melting temperature values of the PVA/Cs blend. ATR-FTIR spectra detected an irregular shift (either red or blue) in some of the characteristic bands of the PVA/Cs nanocomposite, indicating the existence of intra/intermolecular hydrogen bonding creating an interaction between the OH groups of PVA/Cs and ZnO nanoparticles. A thermogravimetric (TGA) analysis demonstrated that the nanocomposites achieved better thermal resistance than a pure PVA/Cs blend and its thermal stability was enhanced with increasing concentration of ZnO nanoparticles. UV analysis showed that with an increase in the content of ZnO NPs, the optical bandgap of PVA/Cs was decreased from 4.43 eV to 3.55 eV and linear and nonlinear parameters were enhanced. Our optical results suggest the use of PVA/Cs/ZnO nanocomposite films for various optoelectronics applications. PVA/Cs/ZnO nanocomposites exhibited significant antibacterial activity against Gram-positive and Gram-negative bacteria. It was found that nanocomposite samples were more effective against Gram-positive compared to Gram-negative bacteria.

## 1. Introduction

Bio-nanocomposites are a promising material due to their remarkable properties and thus have found wide applications in food preservation, packaging, drug delivery, organic dye removal, tissue engineering, and photonics [1]. Recently, the incorporation of nanoparticles into polymer matrices has attracted considerable attention due to the remarkable improvement in the physicochemical properties of nanocomposites even at low levels of nanofillers. Therefore, polymers, especially natural nano-biopolymers, have attracted much attention in recent years [2,3].

Polymer nanocomposites are mainly prepared by adding small amounts of inorganic filler into the host matrix of polymeric materials, while polymer blending involves two or more polymers being mixed to produce novel polymeric materials with promising properties for modern applications. In general, PVA and chitosan have many unique features compared to other polymers, such as biocompatibility, water solubility, non-toxicity, environmental friendliness, and biodegradability [4]. The hydroxyl group (-OH) attached to the mainchain of PVA acts as a source of hydrogen bonding that improves the complexation processes and consequently assists blending with other polymers [5]. Chitosan (Cs) is chitin’s primary derivative, containing 2-amino-2-deoxy-D-glucose and 2-acetamido-2-deoxy-D-glucose. The amine (-NH2) and hydroxyl (OH) groups in the mainchain of Cs facilitate the complexation process with inorganic fillers [6]. Cs has a wide range of medical applications owing to its unique biocompatibility, antimicrobial, and antifungal properties [7,8]. The high density of the reactive functional groups and hydrophilic properties make both PVA and Cs favorable for cross-linking with dopant materials [9,10]. Blending PVA and Cs yields a novel host for different types of dopant materials. The polymer blends can be classified as a completely miscible or compatible polymer blend, or an immiscible or incompatible blend. Adding inorganic particles to mixtures improves various technological properties and reduces cost [11,12].

Zinc oxide (ZnO) nanoparticles have attracted much attention due to their high stability, good photocatalytic activity, antibacterial properties, and nontoxicity [13]. ZnO nanoparticles can be prepared by various techniques, including electrochemical deposition, thermal evaporation, hydrothermal synthesis, and sol-gel [13,14]. Nanoparticles incorporated into the matrix of a polymer blend alters its physicochemical properties due to the interfacial interactions of the polymer blend components with the NPs, as well as the phase morphology of the polymer blend [15,16]. The kinetic parameters of the thermal degradation of polymers is of greater interest due to the improvement of their mechanical and thermal properties [17,18]. It has been reported that the thermal stability of polymers is significantly improved with incorporated metal oxide nanoparticles [19]. 

ZnO nanoparticles have been incorporated into poly (vinyl alcohol)/chitosan polymer blends to enhance their functional properties and antimicrobial activity [20,21,22]. However, incorporating ZnO nanoparticles in a poly (vinyl alcohol)/chitosan polymer blend faces several challenges. One challenge is achieving a homogeneous dispersion of the nanoparticles in the polymer matrix. This is important for enhancing the mechanical properties of the blend films [20,23]. Another challenge is maintaining the stability and compatibility of the nanoparticles with the polymer blend. The addition of ZnO nanoparticles can affect the thermal stability of the blend, and it is important to ensure that the nanoparticles do not degrade or react with the polymer components [23]. Additionally, controlling the concentration of ZnO nanoparticles is crucial to achieve the desired properties of the blend films. Too high of a concentration can led to agglomeration and negatively impact the mechanical properties [24]. Therefore, optimizing the concentration, dispersion, and compatibility of ZnO nanoparticles in the poly (vinyl alcohol)/chitosan polymer blend is essential to overcome this challenge and achieve the desired properties for the desired application, which has been attempted in this work. The effects of ZnO NP content on the structure, chemical composition, thermal degradation, and linear/nonlinear optical properties of a poly (vinyl alcohol)/chitosan polymer blend have been investigated. 

In this work, a simple and environmentally friendly polymer solution blending method was carried out to prepare PVA/Cs/ZnO nanocomposite films with different concentrations of ZnO NPs. The PVA/Cs/ZnO nanocomposite films were investigated with various techniques, such as XRD, ATR-FTIR, DSC, TGA, and UV-vis spectroscopy, and their antibacterial activity was evaluated.

## 2. Experimental Work

### 2.1. Materials

PVA with a molecular weight of 72,000 was supplied by Merck (Rahway, NJ, USA); chitosan (Cs) with a molecular weight of Mw = 60,000–100,000 Da and deacetylation degree of 85% was supplied by Sigma-Aldrich (St. Louis, MI, USA). Zn (NO_3_)_2_ with Mw = 189.4 was supplied by Merck (Rahway, NJ, USA). Acetic acid and NH3 were supplied by Sigma-Aldrich (St. Louis, MI, USA).

### 2.2. Preparation Method

In the current work, the zinc oxide nanoparticles were prepared using co-precipitation. Typically, nitrate Zn (NO_3_)_2_ is dissolved in DI water to form transparent solutions with 0.05 M concentration. The mixture pH was adjusted by dropping 3 mL of NH_3_ solution slowly, followed by stirring for 2 h, till a white precipitate was formed in the solution. The white precipitate was filtered and cleaned several times with DI water to remove any remaining byproducts. The product was dried at 60 °C for 24 h, and later calcined at 110 °C in the oven for 24 h. 

Preparation of PVA/Cs blend: both PVA and chitosan are typically dissolved separately to form individual solutions. In this experiment, 1 g of chitosan powder was dissolved in 200 mL of DI water and acetic acid in a glass beaker. Aqueous solution of polyvinyl alcohol (200 mL) with acetic acid was also prepared. The two solutions were then mixed and stirred for 30 min with a blend ratio 1:1 to obtain a PVA-chitosan blend. To obtain the PVA-Chitosan-ZnO nanocomposite films, 0.3 g of ZnO NPs was dissolved in 40 mL of ethanol and stirred ultrasonically for 30 min. Later, the ZnO solution was mixed with PVA-Chitosan blend with various ratios of ZnO NPs. The PVA-Chitosan-ZnO nanocomposite solution was ultrasonicated to ensure uniform distribution, then poured into a glass substrate and kept for 48 h at 50 °C in an oven. The PVA/Cs blend was prepared with doping of 2 wt%, 5 wt%, 10 wt%, and 15 wt% of ZnO NPs, respectively.

### 2.3. Characterization Methods

Measurements of XRD patterns were performed by a Bruker D8 advance powder XRD with a CuK_α_ radiation source, with λ of 1.5418 Ǻ (I = 50 mA and V = 40 kV). The rate of scanning was 3°/min in the range of 2θ from 40 to 70°. ATR-FTIR spectra were recorded at room temperature in ambient air in the wavenumber range from 4000 to 400 cm^−1^ using a Thermo Scientific iD5 ATR spectrometer (Thermo Electron Scientific Instruments LLC, Madison, WI, USA) with a spectral resolution of 1 cm^−1^. DSC measurements were performed using a Netzsch DSC 214 (NETZSCH-Gerätebau GmbH, Wittelsbacherstrasse, Selb, Germany). All the samples were sealed in an aluminum pan and scans of DSC were performed in the temperature range from 25 to 200 °C with a heating rate of 10 °C/min. The thermal degradation of PVA/Cs and PVA/Cs/ZnO nanocomposites were carried out using a Netzsch TG 209 (NETZSCH-Gerätebau GmbH, Wittelsbacherstrasse, Selb, Germany) Thermogravimetric analyzer (TGA) under a nitrogen atmosphere with a constant flow rate of 20 mL min^−1^ in the temperature range from 25 to 1000 °C at a rate of 10 °C/min. The sample holder used was crucibles made of alumina (Al_2_O_3_) in which approximately 5 mg sample was degraded. 

### 2.4. Antibacterial Activity

The antibacterial activity of PVA/Cs and PVA/Cs/ZnO nanocomposites was evaluated against (*S. aureus*) and (*E. coli*) bacteria with the agar well diffusion method by evaluating the zone of inhibition (mm). This method was reported previously in more detail [3,4].

## 3. Results and Discussion

### 3.1. XRD

**Figure 1:**
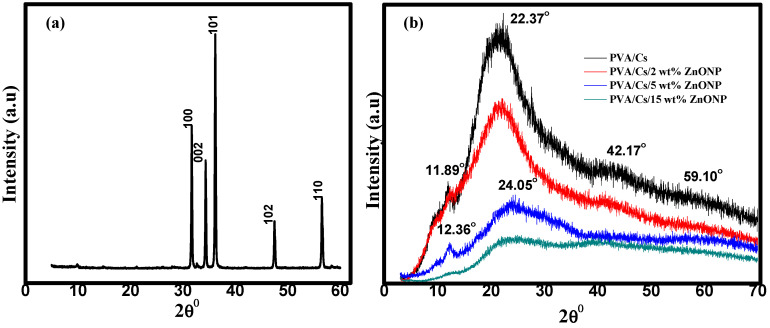
XRD of (**a**) ZnO NPs and (**b**) PVA/Cs and PVA/Cs/ZnO NPs composites.

Figure 1a shows that the XRD pattern of ZnO NPs has several sharp diffraction peaks at 31.69°, 34.37°, 36.32°, 47.53°, and 56.70°. These peaks correspond to the (100), (002), (101), (102), and (110) planes of typical hexagonal wurtzite crystalline ZnO with the space group P63mc (Card No. 96-900-4180) [20]. Figure 1b illustrates the XRD pattern of the PVA/Cs polymer blend and PVA/Cs/ZnO nanocomposites. The XRD pattern of the PVA/Cs polymer blend displayed two diffraction peaks at 2θ = 11.89° and 22.37°. The broad diffraction peak at 2θ = 22.37° confirms the semicrystalline nature of the pure PVA/Cs polymer blend due to the inter/intramolecular interaction through hydrogen bonding among the chains of both PVA and chitosan. On the other hand, we found that with the increase in ZnO NP content in the PVA/Cs blend, the broadness of the diffraction peaks increased, and their intensity decreased, which indicates that the amorphous structure of the PVA/Cs polymer blend was enhanced. The characteristic peaks of the PVA/Cs blend were shifted and observed at 12.36° and 24.05° in PVA/Cs/ZnO nanocomposites. The broadness and decrease in the intensity may be ascribed to the interaction between ZnO NPs and the PVA/Cs polymer blend. 

These results confirm that there is a variation in the electrostatic interactions between PVA/Cs and the ZnO NPs which alters the blend structure with the change in the content of ZnO, causing an increase in the amorphous degree in the nanocomposite samples [24]. Similar behavior has been reported previously for polymeric nanocomposites [25,26,27]. The XRD pattern of nanocomposites with higher ZnO content clearly showed some characteristic diffraction peaks of the ZnO nanoparticles at 2θ = 42.17 and 59.10°, and the major diffraction peaks of the PVA/Cs blend confirmed ZnO nanoparticle incorporation in the nanocomposite’s samples. These characteristic peaks are attributed to the hexagonal Wurtzite structure of ZnO NPs [20]. The observed variation in peak positions and intensities is attributed to the differences in the size of the embedded ZnO nanoparticles and to the lattice strain which is developed in the nanocomposite samples due to the existence of the ZnO NPs [28]. Thus, XRD data confirmed ZnO-NP dispersion in the PVA/Cs polymer blend matrix.

### 3.2. ATR-FTIR Spectra

**Figure 2:**
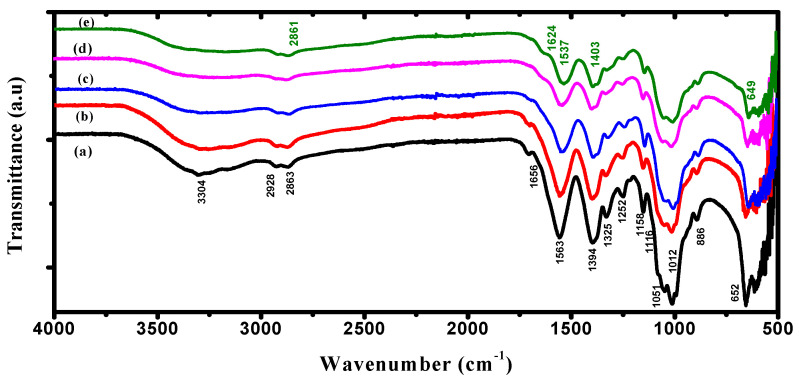
ATR-FTIR spectra of (a) PVA/Cs, (b) PVA/Cs/2 wt% ZnO, (c) PVA/Cs/5 wt% ZnO, (d) PVA/Cs/10 wt% ZnO and (e) PVA/Cs/15 wt% ZnO.

ATR-FTIR spectra were performed to understand the chemical and structural nature of PVA/Cs/ZnO nanocomposites. Figure 2 illustrates the ATR-FTIR spectrum of the PVA/Cs blend and PVA/Cs/ZnO nanocomposites. The spectra of FT-IR confirmed the interaction between metal oxide and polymeric materials in the wavenumber range 500–4000 cm^−1^. The characteristic FT-IR spectrum of the PVA/Cs blend is depicted in Figure 2a. The intense and broad transmission band centered at 3304 cm^−1^ was assigned to the stretching vibration of the –OH and –NH_2_ groups and to O–H/N–H interacting with the oxygen of the C=O group [22]. The bands at 2928 and 2863 cm^−1^ were attributed to the asymmetric stretching vibration of CH_3_ and CH_2_ of chitosan [29]. The bands at 1656 cm^−1^ were ascribed to C=O stretching (Amide I), 1563 cm^−1^ to N-H bending (Amide II), and 1394 cm^−1^ to –COO- stretching vibration. The bands at 1325 cm^−1^ were ascribed to C–O stretching and 1252 cm^−1^ to O–H bending [30,31]. The transmission bands at 1158, 1051, and 1012 cm^−1^ were attributed to the glycosidic linkage and C–O–C vibration of the glucose ring [32]. The band at 1116 cm^−1^, which was observed as a minor shoulder, was attributed to β(1–4) glucosidic stretching [33]. The band at 886 cm^−1^ was ascribed to unsaturated CH_2_ stretching of PVA [34].

Figure 2b–e shows the FT-IR spectra of the PVA/Cs/ZnO nanocomposite samples. It was found that ZnO NPs had a significant effect on some of the functional groups of the PVA/Cs blend due to the complexation or interactions between these groups and ZnO NPs. It was observed that the transmittance of some functional groups was predominately decreased. The intensity of O–H/N–H stretching was found to decrease with increasing ZnO NPs content in the PVA/Cs matrix and became broader and covered a wide range from 3435 to 3104 cm^−1^ compared to the pure PVA/Cs blend, as can be seen in Figure 2e. This can be explained based on the reduction in hydrogen bonds between the –OH and –NH_2_ groups with the incorporation of ZnO NPs into the matrix of the PVA/Cs blend [35]. The band of asymmetric stretching vibration of CH_2_ was slightly red-shifted with a shift order of 2 cm^−1^ and appeared at 2861 cm^−1^ in the highly doped sample of the PVA/Cs blend. The band intensities of amide I and amide II were decreased and red-shifted to a lower wavenumber to appear at 1524 cm^−1^ and 1537 cm^−1^ with shift orders of 32 cm^−1^ and 26 cm^−1^, while the band of COO- stretching vibration was blue-shifted to a higher wavenumber and appeared at 1404 cm^−1^ with a shift order of 10 cm^−1^. These features are because of the strong interaction between the PVA/Cs blend and ZnO NPs by making co-ordinations between these functional groups and Zn^2+^ ions. This is evidence for the hydrogen bond formation between ZnO NPs and the pure PVA/Cs blend. Hence, it can be concluded that ZnO NPs will be located between the PVA/Cs chains which are linked through functional groups [36,37]. The band at 652 cm^−1^ is due to the attachment of the stretching mode of ZnO and the amide group [38]. The bands between 500 and 750 cm^−1^ are attributed to the Zn–O vibration of ZnO [39]. The wavenumber and assignment of the ATR-FTIR bands of PVA/Cs/ZnO nanocomposites are listed in Table 1.

### 3.3. DSC Measurements

**Figure 3:**
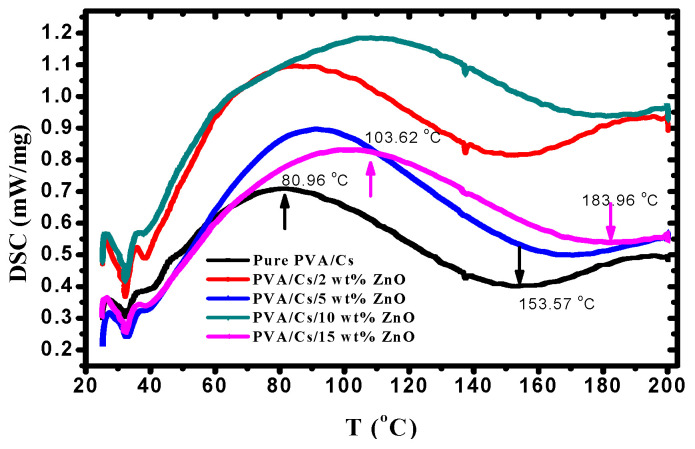
DSC thermograms of PVA/Cs blend and PVA/Cs/ZnO nanocomposites.

Thermal properties of PVA/Cs/ZnO nanocomposites were studied using DSC to interpret the influence of ZnO NPs on the glass transition temperature (T_g_) of PVA/Cs/ZnO nanocomposite samples. DSC thermograms of all samples are displayed in Figure 3 and the results are summarized in Table 2. It is noted that the incorporation of ZnO nanoparticles increased the T_g_ value of the PVA/Cs blend from 80.96 °C to 103.62 °C. The glass transition process was affected by the chain stiffness and molecular packing [40]. The increase in T_g_ values with the increase in ZnO content may be due to the confinement effects and the strong coupling between the PVA/Cs blend and ZnO nanoparticles. This coupling leads to the restriction of the segmental motion of the molecular chains and hence the increase in T_g_. 

The increase in the values of T_g_ of the PVA/Cs blend is similar to the observed results in silica/ PVA composites [41]. It was found that the melting temperature (T_m_) of the PVA/Cs blend was 153.42 °C. Table 2 shows the increase in T_m_ from 153.42 °C to 183.96 °C with the increase in the content of ZnO NPs. The increase in the melting temperature of PVA/Cs/ZnO nanocomposites may be attributed to the strong interactions among hydroxyl groups of the PVA/Cs blend and ZnO NPs.

### 3.4. TGA 

**Figure 4:**
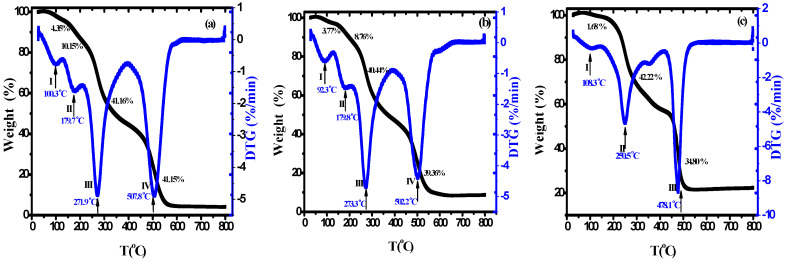
TGA/DTG of (**a**) PVA/Cs, (**b**) PVA/Cs/2 wt% ZnO, and (**c**) PVA/Cs/15 wt% ZnO nanocomposites.

The thermal properties of nanocomposite materials are among the most important properties that are used in packaging applications, particularly in food packaging. TGA is a very important tool for assessing the thermal stability of manufactured nanocomposite materials. Figure 4a–c displays the (TGA) and DTG plots of the PVA/Cs blend and PVA/Cs/ZnO nanocomposites in the temperature range from 25 to 800 °C. It can clearly be seen that the thermal degradation of pure PVA/Cs and nanocomposites with low ZnO NPs content was achieved in four steps, while for nanocomposites with high ZnO NP content it was achieved in three steps. It has been reported that the addition of ZnO to different polymer matrices leads to degradation or stabilization effects [42]. In the first stage, the weight loss for all samples was attributed to the evaporation of water in all samples at different maximum temperatures (T_m_) depending on the content of ZnO NPs in the nanocomposite samples. The water absorption in the PVA/Cs blend is related to the availability of hydroxyl and amino groups that interact with water molecules through hydrogen bonding [43].

**Figure 5:**
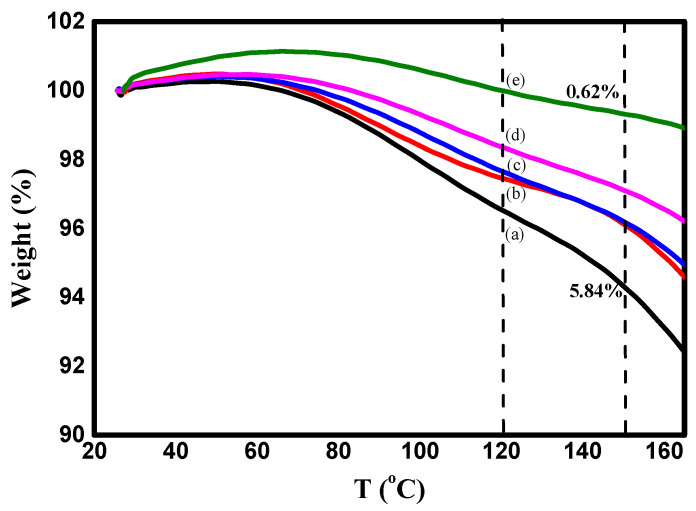
TGA plots of PVA/CS and PVA/Cs/ZnO nanocomposites in the first stage I: (a) PVA/Cs, (b) PVA/Cs/2 wt% ZnO, (c) PVA/Cs/5 wt% ZnO, (d) PVA/Cs/10 wt% ZnO and (e) PVA/Cs/15 wt% ZnO.

The water molecules interacted with various polar groups and the interaction with the amine groups was weaker than those containing hydroxyl groups. It was found that the water content depends on the concentration of ZnO NPs, as shown in Figure 5, and decreases with increasing content of ZnO NPs (5.84% in pure PVA/Cs blend and 0.62% in the highly doped PVA/Cs/ZnO nanocomposite sample at a temperature of 150 °C). Hence, with increasing ZnO NP concentration, a decrease in the water absorption ability was observed.

The weight loss of the PVA/Cs blend and PVA/Cs/ZnO nanocomposites in the second and third stages may be attributed to the decomposition of the chains into small fragments and degradation of saccharide rings [44]. Further weight loss in the fourth stage may be attributed to the cleavage of the C–C bond in the PVA/Cs chain [45]. Pyrolysis of the polysaccharide polymers begins with a random splitting of the glycosidic bond followed by further hydrolysis, forming butyric and acetic acids and a series of lower fatty acids [10]. Maximum temperature ™ with weight loss rate for each degradation stage is a good indicator to define the difference in degradation temperatures of the samples quantitatively. The maximum temperature (*T_m_*), the percentage of weight loss (%), initial temperature (*T_i_*), and the residual mass (%) values for PVA/Cs and PVA/Cs/ZnO nanocomposites in each degradation stage are summarized in Table 3. The initial temperature (*T_i_*) is the temperature at which the loss in weight of the initial sample is 5%. Both the initial temperature and the residual mass of PVA/Cs/ZnO nanocomposites increased with the increase in ZnO NP content, indicating that the nanocomposite samples were more thermally stable than the pure PVA/Cs blend. The same behavior has been reported previously by other researchers [46,47].

In general, the TGA plots revealed that the weight loss of PVA/Cs/ZnO nanocomposites was lower than that of the PVA/Cs polymer blend. The incorporation of ZnO NPs increased the thermal stability of the PVA/Cs matrix, demonstrating that thermal stability is directly related to the concentration of nanoparticles. The change in thermal stability of PVA/Cs/ZnO nanocomposites could be interpreted as follows: on one hand, the existence of ZnO in the PVA/Cs blend may restrict the polymer chains’ mobility due to the formation of networks of polymer chains and inorganic moiety and may act as a thermal insulator and barrier to the volatile products formed during the decomposition process, thus delaying the thermal decomposition [47]. On the other hand, ZnO as a semiconductor can create oxygen and oxygen vacancies via thermal stimulation in the lattice structure. Free oxygen increases the formation of peroxy radicals to break the chains of the polymer and oxygen vacancies will trap and bind electrons to create active catalytic positions in ZnO. Thus, the presence of oxygen and free oxygen vacancies will have a major effect on the decomposition process of polymers [14].

#### Thermal Degradation Kinetics

The TGA/DTG technique is very important in determining the decomposition steps, decomposition temperature, and kinetic parameters of solids. Kinetics of thermal degradation processes are expressed by different equations taking into consideration the characteristic features of its mechanisms. The reaction rate can be described by conversion degree (*g*) based on the following equation [47]:

(1) $$g=\frac{m_{i}-m_{t}}{m_{i}-m_{f}}$$

where *m_i_*, *m_t_*, and *m_f_* are the initial mass, current mass of the sample *t*, and final mass, respectively. The rate of many condensed phase reactions can be determined in terms of the temperature (*T*) and the reaction conversion (*g*). The reaction rate in the non-isothermal kinetics can be expressed as follows:

(2) $$\begin{array}{cc} \frac{dg}{dt}\; & =q\frac{dg}{dT}=k(T)f(g)\\ & =f\exp\left(-\frac{E_{a}}{RT}\right)f(g) \end{array}$$

where *q*, *k*(*T*), *f*(*g*), *f*, and *E_a_* are the linear rate of heating rate, rate constant, differential conversion function, frequency factor, and activation energy and R is the universal gas constant, respectively. The frequency factor based on the Eyring rate theory can be described as follows [48]:

(3) $$f=\frac{\gamma \;ek_{B}T_{m}}{h}\exp\left(\frac{\Delta S}{R}\right)$$

Here, *γ*, *e*, *k_B_*, *T_m_*, *h*, and Δ*S* are the transmission coefficient (unity for monomolecular reaction), Neper number (*e* = 2.7183), Boltzmann’s constant, the maximum temperature of the decomposition stage, Planck’s constant, and the activation of entropy, respectively. Thus, the rate constant can be expressed as follows [49]:

(4) $$k(T)=\frac{\gamma \;ek_{B}T_{m}}{h}\exp\left(\frac{\Delta S}{R}\right)\exp\left(-\frac{E_{a}}{RT}\right)$$

The change in the activation of entropy (Δ*S*) can be estimated using the following equation [50]:

(5) $$\Delta S=2.303\;R\log\left(\frac{fh}{\gamma \;ek_{B}T_{m}}\right)$$

and

(6) $$\Delta H=E_{a}-RT_{m}$$

Hence, from Equations (5) and (6), the Gibbs free energy (Δ*G*) of every stage can be calculated at the maximum temperature (*T_m_*) by a well-known thermodynamic equation as follows:

(7) $$\Delta G=\Delta H-T_{m}\Delta S$$

Activation energy (*E_a_*) and frequency factor (*f*) of each decomposition stage can be evaluated by applying the Coats and Redfern approximation as follows [51]:

(8) $$\log\left[-\frac{\log\left(1-g\right)}{T^{2}}\right]=\log\frac{fR}{qE_{a}}-\frac{E_{a}}{2.303\;RT}+\log\left(1-\frac{2RT}{E_{a}^{}}\right)$$

In the case of *2RT/E_a_* << 1, Equation (8) will become:

(9) $$\log\left[-\frac{\log\left(1-g\right)}{T^{2}}\right]=\log\frac{fR}{qE_{a}}-\frac{E_{a}}{2.303RT}$$

**Figure 6:**
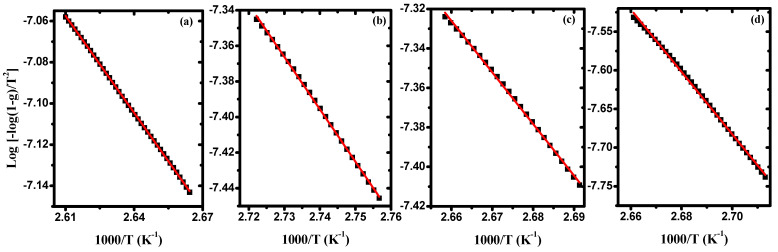
Log[−log(1−g)/T^2^] against 1000/T in the first stage for (**a**) PVA/Cs, (**b**) PVA/Cs/2 wt% ZnO -NPs, (**c**) PVA/Cs/5 wt% ZnO - NPs, and (**d**) PVA/Cs/10 wt% ZnO -NPs.

Figure 6 illustrates the variation of $\log\left[-\frac{\log(1-g)}{T^{2}}\right]$ versus 10^3^/T for pure PVA/Cs and some samples of PVA/Cs/ZnO nanocomposites in the first stage as a representative curve for the other stages. From the intercept and slope, the values of frequency factor, activation energy, and regression of each degradation stage were evaluated for all samples and are presented in Table 4. Also, using Equations (5)–(7), the values of ΔS, ΔH, and ΔG are calculated and given in Table 4. Our results are in good agreement with previously reported data [52,53]. The slight difference between our results and the reported data may be due to different conditions such as the different gas flow and heating rates. Low activation entropy values indicate that the polymer sample has undergone some form of chemical or physical rearrangement of the initial structure, bringing it into thermodynamic equilibrium. This is why the material showed little reactivity in this case, which increases the time required to form the activated complex. In contrast, when the activation entropy values are high, this indicates that the polymer material will not be in thermodynamic equilibrium. The reactivity in this case will be high, and the system will react quickly to produce an active complex; hence, short reaction times are obtained. The negative values of ΔS as mentioned in Table 4 confirm that the formation of the activated complexes is directly related to the decrease in entropy, i.e., the activated complexes are more ordered structures compared to the starting materials, and hence these reactions are classified as slow [49].

**Figure 7:**
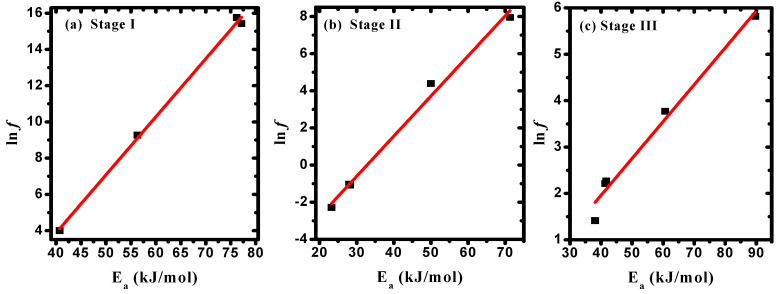
Frequency factor versus the activation energy in (**a**) stage I, (**b**) stage II, and (**c**) stage III.

**Figure 8:**
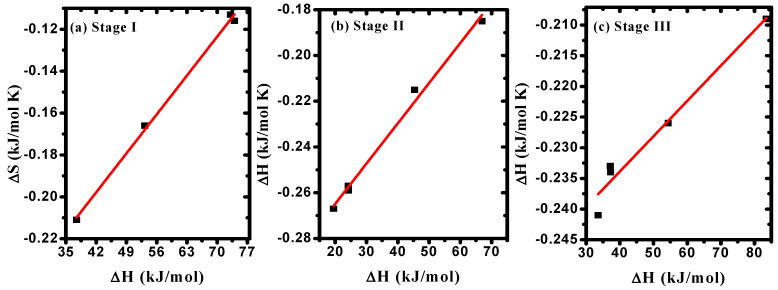
ΔS versus ΔH in (**a**) stage I, (**b**) stage II, (**c**) stage III.

The correlation between *E_a_* and *f* values, as given in Table 4, confirmed the existence of the compensation phenomenon or the isokinetic effect [50], as illustrated in Figure 7a–c. The observed linear relationship between (ΔS) and (ΔH) indicated the existence of the compensation phenomenon in PVA/Cs/ZnO nanocomposites, as shown in Figure 8a–c. Such behavior has been reported for various polymer blends and polymer composites [54,55,56]. Structural changes that may arise when increasing the temperature of the polymeric material to reach equilibrium is the reason for the existence of the linear relationship between ΔH and ΔS, i.e., the variation in ΔH values is compensated by the variation in ΔS values [57]. 

### 3.5. UV-Vis Spectroscopy

**Figure 9:**
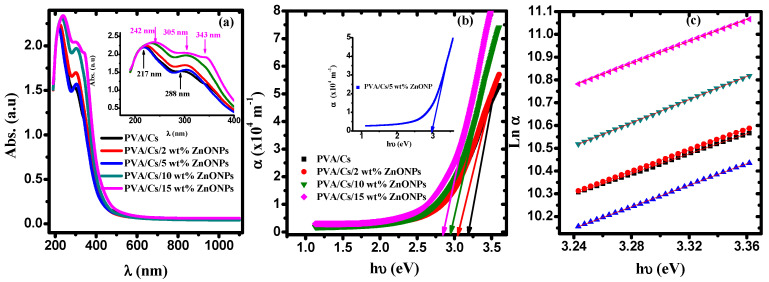
(**a**) The absorption against λ, (**b**) α against hυ, (**c**) Ln α against hυ.

Figure 9a displays the absorption spectra of the PVA/Cs blend and PVA/Cs/ZnO nanocomposites. The spectra of the PVA/Cs blend show an absorption band at 217 nm and a shoulder centered at 288 nm. These bands are attributed to π-π^*^ and to n-π^*^ transition, respectively [58]. These bands shifted after incorporating ZnO NPs and appeared at 242 nm and 305 nm, and a new absorption peak characteristic to ZnO nanoparticles was observed at 343 nm in the highly doped PVA/Cs/ZnO nanocomposite samples [59]. These observations are an indication of the complexation between the ZnO NPs and PVA/Cs matrix by hydrogen bonding through OH groups. Also, the absorption edge was found to be red-shifted to higher wavelengths (lower energies) by increasing the content of ZnO nanoparticles in the PVA/Cs/ZnO nanocomposites.

The values of the absorption edges were estimated from the plot of absorption coefficient (*α* = 2.303 *A/d*; *A* is the absorption and *d* is the thickness of the samples) against the incident photon energy (*h*υ), as shown in Figure 9b. By extrapolating the linear portion of Figure 9b to α = 0 on the x-axis, values of the absorption edge are estimated and given in Table 5. The observed shift in the absorption edge to lower energies (higher wavelengths) with increasing the content of ZnO in PVA/Cs nanocomposites could be attributed to the phase transition, i.e., from the crystalline phase to the amorphous phase, which ultimately may reflect in the optical bandgap energy values. This shift is an indication of the existence of intra-/intermolecular interaction among Zn^+^ ions and adjacent OH groups in the PVA/Cs matrix. 

#### 3.5.1. Urbach Energy (*E_U_*)

More information regarding the band structure of nanocomposite samples can be obtained by knowing the Urbach energy (*E_U_*), which describes the system order of the material by estimating the tail width of localized states in the forbidden gap using the following equation [60]:

(10) α=α0exphυEU

where α_0_ is a pre-exponential factor. Variation of Ln α versus (*h*υ) is shown in Figure 9c. By knowing the slope of the fitted lines in the plot, *E_U_* values are evaluated and tabulated in Table 5. The variation of the *E_U_* values with various concentrations of ZnO NPs is an indication of the enhancement of the amorphous phase of the PVA/Cs matrix and suggested that the trap states are generated because of the doping of ZnO NPs. Moreover, more information regarding the density of defects (DOF) and relaxation of the distorted bond can be obtained by determining the steepness parameter (β) and the electron–phonon interaction strength (*E_e-p_*). Values of β and *E_e-p_* are evaluated as follows [61]:

(11) β=kBTEUEe−p=23β

where *k_B_* and *T* are Boltzmann’s constant and room temperature, respectively. The estimated values *β* and *E_e-p_* are given in Table 5. As the steepness parameter decreases, the values of *E_e-p_* are increased. This variation indicates that near the absorption edge, the defect density states increased, confirming that the crystallinity of PVA/Cs decreases with increasing ZnO NPs content. 

#### 3.5.2. Optical Bandgap Energy (*E_g_*)

**Figure 10:**
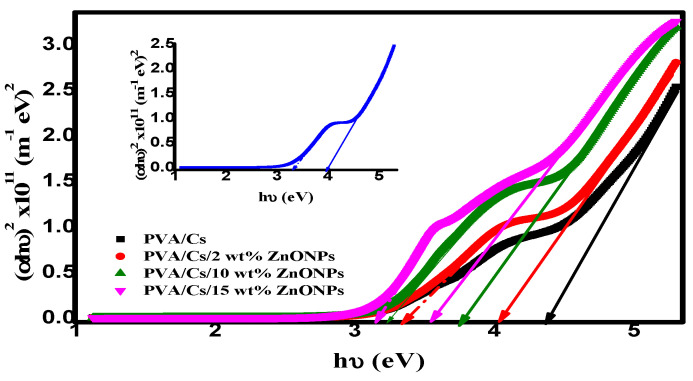
(*αhυ*)^2^ versus *hυ*.

The optical bandgap energy (*E_g_*) of the materials is estimated from Tauc’s equation as follows:

(12) $$\left(\alpha h\upsilon \right)=B{\left(h\upsilon -E_{g}\right)}^{y}$$

where *B* is a constant and correlated with the sample structure and *y* is an empirical index describing the electronic transition. For allowed direct transitions, the *y*-value will be ½, while for allowed indirect transitions, the *y*-value will be ½. Figure 10 shows the variation of (*αhυ*)^2^ against hυ for PVA/Cs with various concentrations of ZnO nanoparticles. By extending the linear part of the plot to intersect the x-axis at (*αhυ*)^2^ = 0, values of direct optical bandgap energy (*E_dg_*) were computed and are given in Table 5. It is evident that as the ZnO NPs content increases, the *E_dg_* values in the nanocomposite samples decrease. It is interesting that there are two values of the direct optical bandgap energies as listed in Table 5. This decrease in *E_dg_* values can be attributed to the generation of new energy levels in the bandgap, which makes it easier for electrons to move from the valence band to the conduction band through these new energy levels. 

The number of carbon clusters (*N_cc_*) of PVA/Cs and PVA/Cs/ZnO nanocomposites was evaluated using the optical bandgap according to the following relation [62]:

(13) Edg=34.4Ncc

The *N_cc_* value was increased from 60 for PVA/Cs to 90 for PVA/Cs/15 wt% ZnO nanocomposite. This increase in the values of *N_cc_* is attributed to the resulting conjugation between monomer units of the PVA/Cs matrix after incorporating the ZnO NPs [63]. The improvement of *N_cc_* values is attributed to the ZnO NP content; more defects in the PVA/Cs matrix are produced with an increase in the content of ZnO, leading to the generation of additional low energy states, and consequently the optical bandgap is decreased, resulting in an improvement of the values of *N_cc_*. 

#### 3.5.3. Refractive Index–Energy Gap Correlation

Many of the optical parameters such as atomic polarizability, static, and high-frequency dielectric constants that contribute effectively to the utilization of materials in optoelectronic applications are directly related to the refractive index (*n*). Also, the refractive index is correlated with the optical energy gap (*E_g_*), and this relationship is the basis for the design of optoelectronic devices. The correlation between the refractive index and the energy gap can be expressed by different relations. A linear relation between the refractive index and the energy gap is proposed by Ravindra et al. as follows [64]:

(14) $$n_{RV}=4.08-0.62E_{g}$$

As the energy levels of the materials can be scaled by a factor of $1/\varepsilon_{opt}^{2}$, where *ε_opt_* = *n^2^*, Moss suggested the following relation [65]:

(15) nM=95Eg0.25

Hervé-Vandamme suggested the following relation according to the theory of vibrations [66]:

(16) $$n_{HV}={\left[1+\left(\frac{E_{H}}{3.4+E_{g}}\right)\right]}^{0.5}$$

where *E_H_* = 13.6 eV represents the ionization energy of hydrogen. Various relations are suggested by Reddy and Singh-Kumar as follows [67,68]:

(17) nRe=ln36.3 Eg−1

(18) $$n_{KS}=KE_{g}^{B}$$

where *K* and *B* equal 3.366 and −0.3223, respectively. The refractive index values of the PVA/Cs blend and PVA/Cs/ZnO nanocomposites were computed based on these different models and are given in Table 6. Obviously, the average value of *n* is enhanced and increased from 1.852 for PVA/Cs to 2.072 for the highly doped nanocomposite sample, indicating that ZnO modified the PVA/Cs matrix and resulted in denser nanocomposites with a lower optical band gap and higher refractive index. Polymeric nanocomposites with higher refractive index values have promising applications in the field of photonics due to their ability to reduce the reflection loss at interfaces and are also used in anti-reflective coating and fabrication of solar cells [69].

#### 3.5.4. Single Oscillator Model

**Figure 11:**
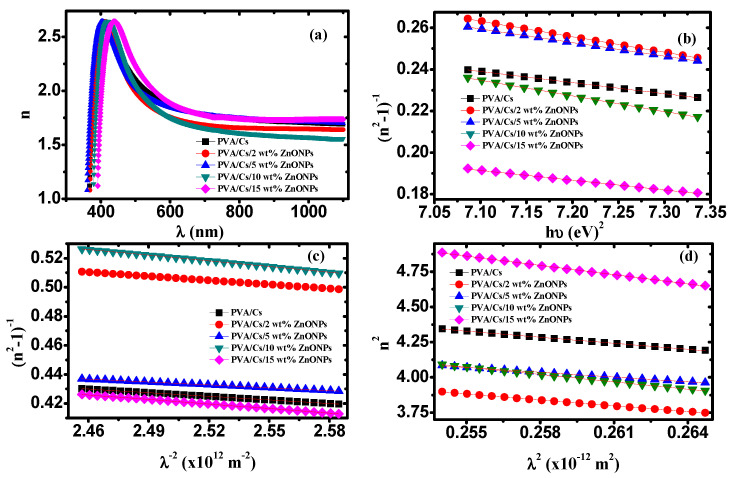
(**a**) *n* versus λ, (**b**) (n^2^ − 1)^−1^ versus (hυ)^2^, (**c**) (n^2^ − 1)^−1^ versus λ^−2^, (**d**) n^2^ versus λ^2^.

Refractive index (*n*) plays an important role in advancing light-emitting diodes, optoelectronics, and waveguides [70]. Values of *n* depend mainly on the bond strength, molecular weight, and density of the material and are related directly to the reflectance (R) and extinction coefficient (*k* = αλ/4π) as follows:

(19) $$n=\left(\frac{1+R}{1-R}\right)+\sqrt{\frac{{\left(1+R\right)}^{2}}{{\left(1-R\right)}^{2}}-{\left(1-k\right)}^{2}}$$

where *R* is estimated using the empirical formula $R=1-\sqrt{T\exp(A)}$ and *T* is the transmittance, respectively [71]. Figure 11a displays the variation of the refractive index against wavelength for all samples. It is observed that the refractive index showed dispersion behavior in the range of 400–600 nm. This dispersion can be interpreted using the single oscillator model (SOM). The correlation between refractive index and photon energy can be presented as follows [71]:

(20) $$\frac{1}{\left(n^{2}-1\right)}=\frac{E_{0}}{E_{d}}-\frac{1}{E_{d}E_{0}}{\left(h\upsilon \right)}^{2}$$

where *E*_0_ and *E_d_* are the oscillation energy and dispersion energy, respectively. The static refractive index (*n_o_*) and static dielectric constant (*ε_s_*) can be estimated using the following equations:

(21) $$n_{0}={\left(1+\frac{E_{d}}{E_{0}}\right)}^{1/2}\;\mathrm{and}\;\varepsilon_{s}=n_{0}^{2}$$

Figure 11b shows the dependence of (n^2^−1)^−1^ on (h*υ*)^2^ for the PVA/Cs blend and PVA/Cs/ZnO nanocomposites. The oscillation and dispersion energy were evaluated by determining the intercept (*E_o_/E_d_*) and slope (*−1/E_o_E_d_*) of the fitted curves of Figure 11b. Also, *n_o_* and *ε_s_* values were estimated using Equation (21) and are given in Table 7. The interaction strengths (*f* = *E_d_E_o_*) between the material and the electromagnetic radiation were also estimated and are listed in Table 7.

The dispersion parameters (*E*_0_ and *E_d_*) were used to calculate the transition moments of the optical spectrum (M_−1_ and M_−3_) of the PVA/Cs blend and the PVA/Cs/ZnO nanocomposite samples as follows [72]:

(22) $$M_{-1}=\frac{E_{d}}{E_{0}}\;\mathrm{and}\;M_{-3}=\frac{M_{-1}}{E_{0}^{2}}$$

The values of *M_−1_* and *M_−3_* are determined and listed in Table 7. *M_–1_* and *M_–_*_3_ values were enhanced with the increase in ZnO nanoparticle content. Since *E*_d_ >> *E*_0_ and the parameters *M_–_*_1_ and *M_–_*_3_ depend mainly on the values of *E_d_* and *E*_0_, *M_–_*_1_ and *M_–_*_3_ values will have similar behavior to *E_d_*. Also, M*_–_*_1_ and *M_–_*_3_ values are increased with decreases in the optical bandgap energy. Since *E*_0_ is proportional to the frequency of optical transition and *E_d_* depends on the charge’s distribution in the unit cell and the chemical bonds confirming the dependence of the optical properties on the material structure, the high values of *E_d_*, *M_–_*_1_, and *M_–_*_3_ indicate that the crystallinity of the PVA/Cs blend is reduced [72]. 

For more information regarding the dispersion of refractive index, a single-term Sellmeier oscillator was applied [73]. Hence, the Wemple–Didomenico formula to calculate the values of *n_o_* at an infinite wavelength should be rewritten as follows [71]:

(23) $$\frac{n_{0}^{2}-1}{n^{2}-1_{}}=1-{\left(\frac{\lambda_{0}}{\lambda }\right)}^{2}$$

where *λ*_0_ is the average oscillator wavelength. Solving for (n^2^ − 1)^−1^ will give

(24) $${\left(n^{2}-1\right)}^{-1}=\frac{1}{\left(n_{0}^{2}-1\right)}-\frac{\lambda_{0}^{2}}{\left(n_{0}^{2}-1\right)}\frac{1}{\lambda^{2}}$$

The dispersion Equation (20) can be rewritten again in terms of *λ*, where E = hc/*λ*, as follows:

(25) $${\left(n^{2}-1\right)}^{-1}=\frac{E_{0}}{E_{d}}-\frac{{\left(hc\right)}^{2}}{E_{o}E_{d}}\frac{1}{\lambda^{2}}$$

Thus, from Equations (24) and (25), we will obtain:

(26) $$\frac{E_{0}}{E_{d}}=\frac{1}{n_{0}^{2}-1}\;\mathrm{and}\;\frac{h^{2}c^{2}}{E_{0}^{}E_{d}}=\frac{\lambda_{0}^{2}}{n_{0}^{2}-1}$$

Consequently, we can obtain the following equations.

(27) $$E_{d}=\frac{hc(n_{0}^{2}-1)}{\lambda_{0}}\;\mathrm{and}\;E_{0}=\frac{hc}{\lambda_{0}}$$

So, Equation (25) can be expressed in terms of (*λ*_0_) and average oscillator strength (*s*_0_) as follows:

(28) $$\frac{1}{\left(n^{2}-1\right)}=\frac{1}{s_{0}\lambda_{0}^{2}}-\frac{1}{s_{0}}\lambda^{-2}$$

Figure 11c illustrates the dependence of 1/(n^2^−1) on *λ*^−2^ for PVA/Cs and the PVA/Cs/ZnO nanocomposites. Values of *s_o_* and *λ_o_* were estimated by determining the slope (−1/*s*_0_) and intercept (1/s_0_ *λ*_0_^2^) of the fitted curves of Figure 11c and are summarized in Table 7. 

The correlation between refractive index and wavelength can be represented using the following equation:

(29) $$n^{2}=\varepsilon_{L}-\frac{e^{2}}{4\pi \varepsilon_{0}c^{2}}\left(\frac{N}{m^{*}}\right)\lambda^{2}$$

where *ε_L_*, *ε*_0_, *e*, *c*, *N*, and m^*^ are the lattice dielectric constant, free space permittivity, electron charge, speed of light, concentration of charge carriers, and electron effective mass, respectively. The dependence of *n^2^* on *λ*^2^ for PVA/Cs and the PVA/Cs/ZnO nanocomposites are displayed in Figure 11d. *ε_L_* and (N/m^*^) values for all samples were evaluated from the intercept and slope of the fitted lines in Figure 11d and are given in Table 7. Values of charge carrier concentration (*N*) were estimated by the effective mass of the electron (m^*^ = 0.44*m_o_*, where *m_o_* is the electron rest mass) and are summarized in Table 7. It was found that the values of (*N*) increased with increasing ZnO content in PVA/Cs. Also, it was found that *ε_L_* values were higher than the values of *ε*_s_ as listed in Table 7. The difference between *ε_L_* and *ε_s_* values was ascribed to the increase in the free carrier concentrations and to the contribution of the polarization which resulted within the material after light fell on the sample [14]. The variation of *E_d_*, *E*_0_, *ε_s_*, and *n*_0_ values indicates an increase in the charge transfer between the PVA/Cs macromolecules and ZnO NPs and an increase in the disorder degree in the PVA/Cs matrix [74,75]. The plasma frequency (*ω_p_*) of PVA/Cs and PVA/Cs/ZnO nanocomposites were calculated based on the Drude free-electron mode according to the following equation and are also summarized in Table 7:

(30) $$\omega_{p}^{2}=\frac{e^{2}}{\varepsilon_{0}}\left(\frac{N}{m^{*}}\right)$$

Equation (30) indicates that *ω_p_* values depend mainly on values of *N*. When the frequency (*ω*) of the incident light and plasma frequency (*ω_p_*) are equal, higher absorption occurs. However, when *ω* < *ω_p_*, no radiation can propagate within the materials while, when *ω* > *ω_p_* the material becomes transparent, and the electronic oscillation occurs.

#### 3.5.5. Linear and Nonlinear Parameters

When a light with high intensity falls on the material, nonlinear behavior is produced. The resulting polarization (*P*) inside the material is a nonlinear function of an electric field (*E*) and can be expressed as follows [76]:

(31) $$\begin{array}{cc} P\; & =\chi^{\left(1\right)}E+P_{NL}\\ & =\chi^{\left(1\right)}E+\chi^{\left(2\right)}E^{2}+\chi^{\left(3\right)}E^{3} \end{array}$$

where *χ*^(1)^, *χ*^(2)^, and *χ*^(3)^ are the first, second, and third orders of optical susceptibility. The linear (*χ*^(1)^) and non-linear cubic order (*χ*^(3)^) susceptibilities can be evaluated using the dispersion parameters (*E*_0_ and *E_d_*) or the static refractive index (*n*_0_) as follows [77]:

(32) $$\chi^{\left(1\right)}=E_{d}/4\pi E_{0}$$

(33) $$\chi^{\left(3\right)}=A{\left(\chi^{\left(1\right)}\right)}^{4}$$

(34) $$\chi^{\left(3\right)}=\frac{A}{{\left(4\pi \right)}^{4}}{\left(n_{0}^{2}-1\right)}^{4}$$

where *A* = 1.79 × 10^−10^ esu. Considering that *χ*^(3)^ is a dominant nonlinear parameter in the materials, it is generated by the excitation below the optical bandgap energy in the transparent frequency region. Also, the nonlinear refractive index (*n*_2_) is evaluated from optical band gap energy using the following equation [78]:

(35) $$n_{2}=\frac{G}{E_{dg}^{4}}$$

where G is a constant equal to 1.26 × 10^−9^ [esu (eV)^4^]. Values of *χ*^(1)^, *χ*^(3)^, and the n_2_ of PVA/Cs and the PVA/Cs/ZnO nanocomposites were determined and are given in Table 8. The values of χ^(1)^, χ^(3)^, and n_2_ show that the PVA/Cs/ZnO nanocomposites can be strongly nominated for use in optical power reduction, optical switching, photonics, fiber communication, and optical information processing [79].

#### 3.5.6. Dielectric Constant and Optical Conductivity

**Figure 12:**
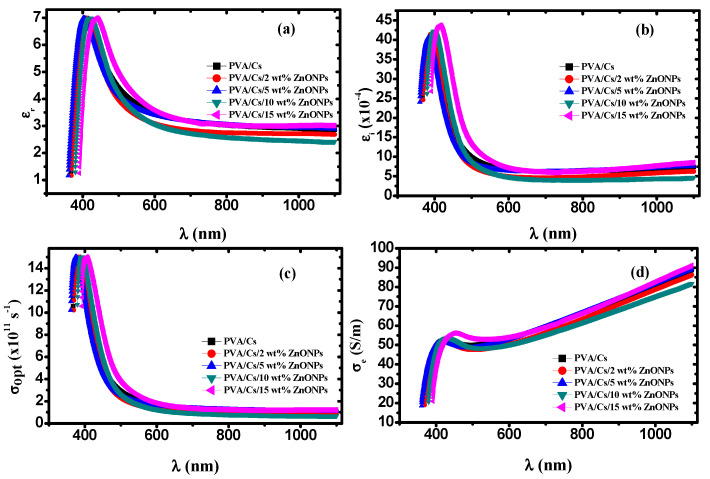
(**a**) *ε_r_* versus λ, (**b**) *ε_i_* versus λ, (**c**) *σ_opt_* versus λ, (**d**) *σ_e_* versus λ.

Figure 12a,b depicts the behavior of the real part ($\varepsilon_{r}=n^{2}-k^{2}$) and imaginary part ($\varepsilon_{i}=2nk$) of the complex dielectric constant ($\varepsilon^{*}=\varepsilon_{r}-i\varepsilon_{i}$) for the PVA/Cs and PVA/Cs/ZnO nanocomposites as a function of wavelength. It was found that both *ε_r_* and *ε_i_* behave with the same trend with the increase in the ZnO content as well as the wavelength. They increased with the increase in the content of ZnO and decreased with increasing wavelength. The higher values of *ε_r_* and *ε_i_* at lower wavelengths are attributed to the high contribution of charge carrier. Also, a wide dispersion region in the behavior of *ε_r_* and *ε_i_* below 600 nm was observed because of the polar nature of nanocomposite samples that follows the fluctuation of the incident field [8,22].

The dispersion regions play an important role in designing optical communication systems. The molecules cannot follow the fluctuations of the incident field at higher wavelengths because of the inertia, and consequently *ε_r_* remains constant [80,81], while at lower wavelengths the high values of *ε_i_* (Figure 12b) are attributed to the dipolar polarization of the materials.

Optical and electrical conductivities (*σ_opt_* & *σ_e_*), which describe the behavior of the conductivity of materials because of charge carrier transport due to changing electric fields of incident electromagnetic waves, can be evaluated by the optical parameters using the following equations [82]:

(36a) $$\sigma_{opt}=\frac{\alpha nc}{4\pi }$$

(36b) $$\sigma_{e}=\frac{2\lambda \sigma_{opt}}{\alpha }$$

Figure 12c,d depicts the dependence of *σ_opt_* and *σ_e_* of PVA/Cs and PVA/Cs/ZnO nanocomposites on the wavelength. Both *σ_opt_* and *σ_e_* behave in different ways. It was found that the value of *σ_opt_* was in the order of 10^12^ s^−1^ in the UV region, which indicates that PVA/Cs/ZnO nanocomposite samples have a high photo response, meaning these nanocomposites are strong candidates for use in information processing. Higher values of *σ_opt_* in the high absorption region at lower wavelengths (higher energies) and higher ZnO nanoparticle content are ascribed to the higher charge carrier density. Moreover, the reduction in the optical bandgap with the increase in the ZnO NP content in the nanocomposites will lead to an increase in the number of energy localized states as well as the charge carrier concentration, resulting in an increase in the values of *σ_opt_*. On the other hand, it was found that *σ_opt_* decreased with increasing wavelength. This behavior in this wavelength region can be attributed to the lack of availability of mobile charge carrier for transition between localized states because of interfacial polarization (IP) [83]. With increasing wavelength, the average displacement of charge carrier is decreased and thus the conductivity will decrease [22,84]. Furthermore, the electrical conductivity was enhanced with an increase in the content of ZnO NPs and found to range from 17 to ~92 S/m, revealing that the PVA/Cs/ZnO nanocomposite has a semiconducting nature.

### 3.6. Antibacterial Activity

**Figure 13:**
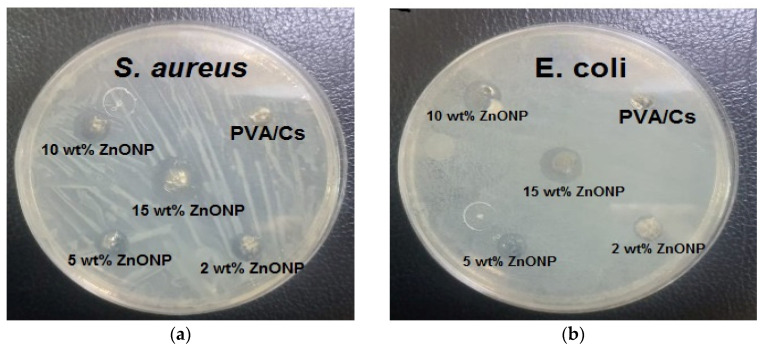
The bacterial inhibition zones of PVA/Cs and PVA/Cs/ZnONPs with different ratios (2, 5, 10, and 15 wt%) against (**a**) *S. aureus* and (**b**) *E. coli*.

**Figure 14:**
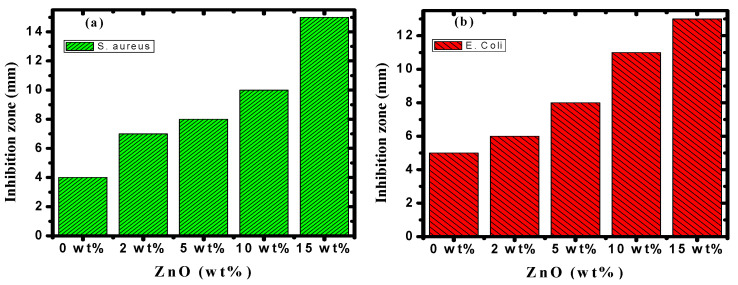
The antibacterial activity of PVA/Cs/ZnO nanocomposites against (**a**) *S. aureus* and (**b**) *E. coli* bacteria.

**Figure 15:**
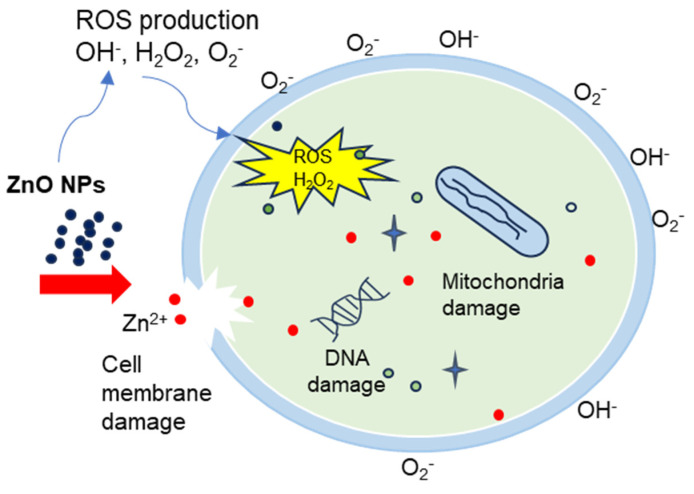
The mechanism representation of ZnO NPs anti-bactericidal activity.

Figure 13 and Figure 14 illustrate the bacterial activity of PVA/Cs/ZnO nanocomposites against Gram-positive (*S. aureus*) and Gram-negative (*E. coli*) bacteria. The Gram-positive (*S. aureus*) bacteria were more strongly affected by both the PVA/Cs blend or by the PVA/Cs/ZnO nanocomposite than the Gram-negative (*E. coli*) bacteria. Due to the existence of teichoic acid in the Gram-positive bacteria as well as phospholipids in the Gram-negative bacteria, the bacterial cell contains a negatively charged membrane [7]. As the PVA/Cs blend is positively charged, it can interact electrostatically with the negatively charged lipidic bacterial membranes and consequently can alter its permeability, ultimately affecting cell growth and viability. Also, since the Gram-positive bacteria (*S. aureus*) have only one cytoplasmic membrane, they will be more susceptible to the effects of surface rupture than the Gram-negative (*E. coli*) bacteria, which have a double outer membrane (a plasma membrane with a peptidoglycan thin layer and an outer membrane) [85]. Also, it is noticeable that the antibacterial activity was improved with an increase in ZnO NP content in the nanocomposite samples. In general, ZnO NPs have a wide spectrum of antibacterial activity through several mechanisms that include reactive oxygen species production (i.e., hydroxyl radicals (OH^−^), hydrogen peroxide (H_2_O_2_), and peroxide (O_2_^−^)) and membrane disruption [86]. The toxicity of reactive oxygen species (ROS) against bacteria can be attributed to its high reactivity and oxidative properties. The toxicity of these products will involve the destruction of most cellular components such as proteins, DNA, and lipids, because of their internalization into the membrane of the bacteria cell. The mechanism of ZnO NPs’ interaction with bacterial cells is represented in Figure 15. Because of the negative charge, the hydroxyl radicals and peroxides cannot penetrate the bacterial membrane and thus remain on the bacteria’s outer surface [87]. In contrast, H_2_O_2_ molecules can pass through the cell wall of the bacteria, which subsequently leads to infections and destruction, and eventually leads to cell death [88]. When ZnO nanoparticles kill or interact with the cell membrane, it is likely that the particles remain strongly adsorbed on the surface of the killed/remaining bacteria, preventing additional antibacterial activity. Once the ZnO nanoparticles are in the growth medium, they will continue to release peroxides covering the entire surface of the dead bacteria. Hence, this continuous release of peroxide will increase the bactericidal efficacy. 

Also, the release of zinc ions (Zn^2+^) causes cell membrane damage and mitochondria to weaken, and restricts the cell growth by leaking DNA, proteins, and lipids, ultimately leading to cell destruction. Released Zn^2+^ has a significant effect on inhibiting active transport as well as in amino acid metabolism and perturbing the enzyme system [89]. Similar results of the antibacterial activity of polymeric nanocomposites against Gram-positive and Gram-negative bacteria were published previously [3,4].

## 4. Conclusions

XRD study showed that by increasing the content of ZnO nanoparticles, the intensity of the main characteristic peaks of the PVA/Cs blend was decreased and shifted to higher diffraction angles, indicating that an interaction between PVA/Cs and ZnO had taken place. ATR-FTIR investigation verified the modifications in the structure of PVA/Cs due to the doping with ZnO. These modifications were observed in the locations and intensities of transmission bands of PVA/Cs/ZnO nanocomposites in comparison with the pure PVA/Cs blend. Analysis of DSC data showed that the values of T_g_ and T_m_ were shifted to higher temperatures by increasing the content of ZnO NPs, and this shift was attributed to the strong interaction between the –OH groups of PVA/Cs and ZnO nanoparticles. TGA revealed that the thermal stability of PVA/Cs/ZnO nanocomposites was enhanced with increasing content of ZnO nanoparticles. The decrease in the optical bandgap from 4.43 eV for PVA/Cs to 3.55 eV for PVA/Cs/15 wt% ZnO nanocomposite was explained in terms of created localized states and defects, as verified by the Urbach energy investigation. Single oscillator and Sellmeier oscillator models were used to investigate the effect of ZnO NPs on the dispersion performance of the PVA/Cs blend. The dielectric constant (*ε_L_*) was greatly improved from 7.98 for PVA/Cs to 10.44 for PVA/Cs/15 wt% ZnO nanocomposite, and the values of (N/m^*^) of PVA/Cs blend were enhanced. The nonlinear parameters of the PVA/Cs blend were also improved and the values of *χ*^(3)^ and *n*_2_ were duplicated after doping with ZnO NPs. These novel results nominate PVA/Cs/ZnO nanocomposites for updated optoelectronic applications. Antibacterial activity of PVA/Cs/ZnO nanocomposites was greatly enhanced against both Gram-positive and Gram-negative bacterial pathogens with an increase in the content of ZnO NPs. Hence, PVA/Cs/ZnO nanocomposites could inhibit bacterial pathogens.

## Figures and Tables

**Table 1:** The wavenumber and assignment of ATR-FTIR bands of PVA/Cs/ZnO nanocomposites.

Wavenumber	Assignment	Ref.
3304	stretching vibration of –OH and –NH_2_ groups	[22]
2928	asymmetric stretching vibration of CH_3_	[29]
2863	asymmetric stretching vibration of CH_2_	[29]
1656	C=O stretching	[30,31]
1563	N-H bending	[30,31]
1394	–COO- stretching vibration	[30,31]
1325	C–O stretching	[30,31]
1252	O–H bending	[30,31]
1158, 1051, 1012	glycosidic linkage and C–O–C glucose ring vibration	[32]
1116	β (1- 4) glucosidic stretching	[33]
886	unsaturated CH_2_ stretching of PVA	[34]
652	O-Zn-O	[38]

**Table 2:** T_g_ and T_m_ values of PVC/Cs and PVC/Cs/ZnO nanocomposite samples.

Sample	T_g_ (°C)	T_m_ (°C)
PVA/Cs	80.96	153.42
PVA/Cs/2 wt% ZnONPs	81.86	153.09
PVA/Cs/5 wt% ZnONPs	91.13	165.02
PVA/Cs/10 wt% ZnONPs	103.61	178.95
PVA/Cs/15 wt% ZnONPs	103.62	183.96

**Table 3:** Maximum temperature (*T_m_*) and weight loss percentage (%) for PVA/Cs/ZnO nanocomposites.

Sample	Stage I		Stage II		Stage III		Stage IV		Residual Mass	T_i_ (°C)
	*T_m_* °C	Weight (%)	*T_m_* °C	Weight (%)	*T_m_* °C	Weight (%)	*T_m_* °C	Weight (%)		
PVA/Cs	100.3	4.19	179.7	10.15	271.9	41.16	507.8	40.61	4.15	142
PVA/Cs/2 wt% ZnO	92.30	3.29	179.8	8.67	273.3	40.44	502.2	39.36	8.72	161
PVA/Cs/5 wt% ZnO	100.6	3.46	207.6	10.50	265.1	40.38	508.8	27.66	8.21	164
PVA/Cs/10 wt% ZnO	108.4	2.76	253.9	47.42	486.4	37.45	---	---	12.37	178
PVA/Cs/15 wt% ZnO	108.3	1.68	250.5	42.22	478.1	34.80	---	---	22.44	210

**Table 4:** The kinetic parameters for PVA/Cs and PVA/Cs/ZnO nanocomposites are based on C–R approach.

Sample	E_a_ (kJ/mol)	r^2^	*f* (Hz)	ΔS (kJ/mol K)	ΔH (kJ/mol)	ΔG (kJ/mol)
Stage I						
Pure PVA/Cs	40.72	1.00000	54.58	−0.211	37.48	116.23
PVA/Cs/2 wt% ZnO	56.29	0.99938	1.06 × 10^4^	−0.166	53.25	114.03
PVA/Cs/5 wt% ZnO	76.20	0.99878	6.99 × 10^6^	−0.113	73.09	115.38
PVA/Cs/10 wt% ZnO	77.16	0.99874	5.01 × 10^6^	−0.116	73.99	118.45
Stage II						
Pure PVA/Cs	23.22	0.99927	0.102	−0.267	19.46	140.77
PVA/Cs/2 wt% ZnO	27.95	0.99984	0.354	−0.257	24.19	140.87
PVA/Cs/5 wt% ZnO	28.24	0.99964	0.341	−0.259	24.25	148.81
PVA/Cs/10 wt% ZnO	49.98	0.99996	81.22	−0.215	45.40	158.88
PVA/Cs/15 wt% ZnO	71.32	0.99990	2.85 × 10^3^	−0.185	66.97	164.16
Stage III						
Pure PVA/Cs	38.10	0.99984	4.11	−0.241	33.57	164.75
PVA/Cs/2 wt% ZnO	41.38	0.99989	9.15	−0.234	37.29	165.20
PVA/Cs/5 wt% ZnO	41.68	0.99974	9.65	−0.233	37.21	162.82
PVA/Cs/10 wt% ZnO	60.67	0.99954	43.41	−0.226	54.36	226.00
PVA/Cs/15 wt% ZnO	89.74	0.99953	335.92	−0.209	83.50	240.36
Stage IV						
Pure PVA/Cs	72.18	0.99863	246.08	−0.212	65.59	231.28
PVA/Cs/2 wt% ZnO	63.79	0.99880	63.31	−0.233	57.35	230.31
PVA/Cs/5 wt% ZnO	20.10	0.99973	0.029	−0.287	13.60	237.90

**Table 5:** Optical parameter values of PVA/Cs/Ag nanocomposites.

Samples	Optical Parameters						
	*E_ed_ (eV)*	*E_U_* (eV)	*β*	*E_e-p_*	*E_dg_*_1_ (eV)	*E_dg_*_2_ (eV)	*Ncc*
PVA/Cs blend	3.19	0.42	0.061	10.92	4.43	---	60
PVA/Cs/2 wt% ZnONPs	3.05	0.39	0.065	10.14	4.03	3.35	73
PVA/Cs/5 wt% ZnONPs	2.89	0.42	0.062	10.74	3.95	3.33	75
PVA/Cs/10 wt% ZnONPs	2.94	0.45	0.057	11.66	3.78	3.25	83
PVA/Cs/15 wt% ZnONPs	2.83	0.43	0.060	11.06	3.55	3.15	94

**Table 6:** Refractive index values based on the different models.

Sample	*E_ed_ (eV)*	*n_RV_*	*n_M_*	*n_HV_*	*n_Re_*	*n_KS_*	*n* *_Average_*
PVA/Cs	3.19	1.333	2.086	1.654	2.103	2.083	1.852
PVA/Cs/2 wt% ZnONPs	3.05	1.581	2.134	1.682	2.198	2.148	1.949
PVA/Cs/5 wt% ZnONPs	2.89	1.631	2.145	1.688	2.218	2.162	1.968
PVA/Cs/10 wt% ZnONPs	2.94	1.736	2.167	1.701	2.262	2.193	2.012
PVA/Cs/15 wt% ZnONPs	2.83	1.879	2.201	1.719	2.324	2.237	2.072

**Table 7:** Dispersion parameter values of PVA/Cs/ZnO nanocomposites.

Parameter	PVA/Cs Blend	PVA/Cs/ 2 wt% ZnONPs	PVA/Cs/ 5 wt% ZnONPs	PVA/Cs/ 10 wt% ZnONPs	PVA/Cs/ 15 wt% ZnONPs
*E*_0_ (eV)	3.41	3.28	3.34	3.20	3.38
*E_d_* (eV)	5.53	4.113	4.61	4.16	6.44
*n*_0_	1.61	1.50	1.54	1.52	1.41
**ε*_s_*	2.62	2.26	2.38	2.30	2.91
*f* (eV)^2^	18.87	13.51	15.38	13.33	21.74
*M_−_*_1_	1.27	1.12	1.18	1.14	1.38
*M_−_*_3_, (eV)^−2^	0.109	0.104	0.105	0.111	0.121
**ε*_L_*	7.98	7.45	6.96	8.51	10.44
N/m^*^(×10^58^ m^−3^ kg^−1^)	1.75	0.91	1.39	2.13	2.68
*N* (×10^27^ m^−3^)	7.04	3.66	5.28	8.55	10.8
*ω*_p_ (×10^15^ Hz)	7.13	5.14	6.34	7.86	8.82
*s*_0_ (×10^13^ m^−2^)	1.20	1.06	1.52	0.76	0.96
*λ*_0_ (×10^−7^ m)	3.62	3.55	3.32	3.92	3.90

**Table 8:** Linear and nonlinear optical parameters values of PVA/Cs/ZnO nanocomposites.

Optical Parameter	PVA/Cs	PVA/Cs/ 2 wt% ZnONPs	PVA/Cs/ 5 wt% ZnONPs	PVA/Cs/ 10 wt% ZnONPs	PVA/Cs/ 15 wt% ZnONPs
*χ* ^1^	0.128	0.100	0.110	0.103	0.152
*χ*^3^ (×10^−14^ esu)	4.93	1.81	2.63	2.05	9.54
*n*_2_ (×10^−12^)	3.27	4.77	5.17	6.17	7.93

## Data Availability

Full experimental data and results are available upon request.
